# Association between *PXR* polymorphisms and cancer risk: a systematic review and meta-analysis

**DOI:** 10.1042/BSR20171614

**Published:** 2018-06-12

**Authors:** Jing Wen, Zhi Lv, Hanxi Ding, Xinxin Fang, Mingjun Sun

**Affiliations:** 1 Department of Gastroenterology, The First Affiliated Hospital of China Medical University, Shenyang 110001, Liaoning, China; 2 Department of Anorectal Surgery, The First Affiliated Hospital of China Medical University, Shenyang 110001, Liaoning, China; 3 Tumor Etiology and Screening Department of Cancer Institute and General Surgery, The First Affiliated Hospital of China Medical University, and Key Laboratory of Cancer Etiology and Prevention (China Medical University), Liaoning Provincial Education Department, Shenyang 110001, Liaoning, China

**Keywords:** Cancer, pregnane X receptor, Polymorphism, Risk

## Abstract

Current studies have explored the correlation between the single nucleotide polymorphisms (SNPs) of pregnane X receptor (*PXR*) and cancer risk. However, the findings were conflicting. Hence, we performed a comprehensive review and meta-analysis for these researches to determine the effect of *PXR* polymorphisms on the risk of cancer. Eligible publications were collected based on a series of rigorous inclusion and exclusion criteria. In consequence, a total of eight case–control studies (from seven citations) covering 11143 cases and 12170 controls were involved in a meta-analysis of ten prevalent *PXR* SNPs (rs10504191 G/A, rs3814058 C/T, rs6785049 A/G, rs1464603 A/G, rs1523127 A/C, rs2276706 G/A, rs2276707 C/T, rs3732360 C/T, rs3814055 C/T, rs3814057 A/C). The correlations between *PXR* SNPs and cancer risk were estimated by odds ratios (ORs) with their 95% confidence intervals (95%CIs). The findings demonstrated that rs3814058 polymorphism (CT compared with CC: pooled OR = 1.280, *P*=6.36E-05; TT compared with CC: pooled OR = 1.663, *P*=2.40E-04; dominant model: pooled OR = 1.382, *P*=2.58E-08; recessive model: pooled OR = 1.422, *P*=0.002; T compared with C: pooled OR = 1.292, *P*=6.35E-05) and rs3814057 polymorphism (AC compared with AA: pooled OR = 1.170, *P*=0.036; dominant model: pooled OR = 1.162, *P*=0.037) were associated with the risk of overall cancer. In stratified analyses, rs3814058 polymorphism was revealed to increase the cancer risk in lung cancer subgroup. In summary, this meta-analysis indicates that the rs3814057 and rs3814058 polymorphisms of *PXR* gene play crucial roles in the pathogenesis of cancer and may be novel biomarkers for cancer-forewarning in overall population or in some particular subgroups.

## Introduction

The pregnane X receptor (*PXR*), also referred to as nuclear receptor subfamily 1 group I member 2 (*NR1I2*) and steroid and xenobiotic receptor (*SXR*), is a ligand-dependent transcription factor belonging to the orphan nuclear receptors superfamily [1–3] and plays an essential role in adaptive defense system against endogenous metabolites and toxic xenobiotics [4]. The discovery of the *PXR* supplied novel perspectives on the molecular basis of the drug resistance in cancer cells [5]. What is more, *PXR* also participates in regulating the proliferation of either cancer or non-cancer cells. In cancer cells, it can control cell growth in various cancer tissues such as ovarian, prostate, colon, endometrial, breast, and so on [6–10]. Strong associations have been revealed between *PXR* and the proliferation of cancer [1,4,11].

The *PXR* gene is located on chromosome 3q12-13.33, spanning 35 kb with ten exons and nine introns. Its coding protein contains a ligand-dependent transactivation function 2, a ligand-binding domain, a hinge region, and a DNA-binding domain [12]. Numerous single nucleotide polymorphisms (SNPs) have been observed in *PXR* gene and the putatively functional SNPs may influence its expression or function. Currently, accumulating studies have yet investigated the associations between SNPs of *PXR* and the cancer susceptibility, however, the findings were conflicting. For instance, the rs3814057 polymorphism was related to an elevated cancer risk in our meta-analysis, while it showed no association in Christina Justenhoven’s study [13]. Additionally, no systematic review containing all tested SNPs of *PXR* has been published yet.

We aim to fill this blank by performing a systematic review and meta-analysis of the available evidence, explore the correlation of *PXR* SNPs with cancer susceptibility, and provide clues for researchers to design future studies and screen novel functional genetic biomarkers for cancer prediction.

## Materials and methods

### Retrieval strategy

A comprehensive literature search was performed independently by two investigators (J.W. and Z.L.) to find all publications regarding the correlation between the *PXR* polymorphisms and cancer risk. We retrieved the PubMed and Web of Science database by using the following query terms: ‘(*PXR* or pregnane X receptor or *NR1I2* or nuclear receptor 1I2 or nuclear receptor subfamily 1 group I member 2 or or *SXR* or steroid X receptor or ‘steroid and xenobiotic receptor’) and (polymorphism or SNP or variant or variation) and (cancer or tumor or carcinoma or neoplasm)’, up to 16 November 2017.

### Inclusion and exclusion criteria

The following inclusion criteria were adopted to identify all eligible publications: (i) a case–control-designed study; (ii) about the association between *PXR* SNPs and cancer risk. The main exclusion criteria were: (i) duplicate studies; (ii) unrelated to cancer or *PXR* SNPs; (iii) not sufficient data.

### Data extraction

Data extraction was independently completed by two of the investigators (J.W. and Z.L.). Items obtained from each eligible publication included: first author, year of publication (unpublished showed study year), country of origin, cancer type, SNP locus, sample size, genotype counts in cases and controls, Hardy–Weinberg equilibrium (HWE) in controls, source of control groups, genotyping method, and adjusted factors. If an article contained multiple study populations or sources, data were extracted respectively. If data were unreported in eligible articles, we spared no effort to contact the corresponding authors.

### Methodology quality assessment

The quality evaluation of the selected studies was scored by two reviewers (J.W. and H.D.) independently, according to a study on assigning quality scores which was mentioned in a previous meta-analysis [14]. A third investigator (X.F.) would be involved when disagreement existed. Six items were evaluated: (i) representativeness of the cases; (ii) source of the controls; (iii) ascertainment of relevant cancers; (iv) sample size; (v) quality control of the genotyping methods; (vi) HWE. The scores for quality assessment ranged from 0 to 10 and studies with less than 5 score were not involved in the subsequent analysis.

### Trial sequential analysis

The results of meta-analysis can be misled by random errors (play of chance) or systematic errors (bias) due to sparse data and/or repeated significance testing. Therefore, a trial sequential analysis tool (TSA from Copenhagen Trial Unit, Center for Clinical Intervention Research, Denmark, 2011) was conducted in our meta-analysis to gain more reliable findings [15]. In brief, TSA evaluates the required information size by setting type-I error of 5%, type-II error of 20%, and statistical test power of 80%, and then plots a two-sided graph, where TSA monitoring boundaries (red lines) were built [16]. If the TSA monitoring boundaries were crossed with Z-curve (blue lines) before reaching the required information size, robust conclusion might have been identified and further studies are unnecessary [16]. Otherwise, more trials are still in demand.

### False-positive report probability

We evaluated the significant findings by computing false-positive report probability (FPRP), which was based on observed *P*-value, statistical power of test, and prior probability [17]. To identify a significant association as ‘noteworthy’, prior probabilities of 0.25, 0.01, 0.001, 0.0001 were assigned and 0.2 was set as FPRP cut-off value [18].

### Statistical analysis

All the statistical analyses in the present study were performed by STATA software, version 11.0 (STATA Corp., College Station, TX, U.S.A.). All tests were two-sided and *P*-value <0.05 was considered as a statistical significance level unless we highlighted once more. The dominant genetic model was defined as homozygote + heterozygote variant compared with homozygote wild, while the recessive genetic model was defined as homozygote variant compared with homozygote + heterozygote wild. The HWE for the genotype distributions of *PXR* SNPs in controls was calculated by chi-square test, and *P*-values <0.05 was considered as significant disequilibrium. The intensity of the relations between the *PXR* SNPs and cancer risk was evaluated by pooled odds ratios (ORs) and 95% confidence intervals (95%CIs), calculated by fixed effect model [19] when the between-study heterogeneity was absent, otherwise random effect models [20]. The between-study heterogeneity was calculated by Cochran’s *Q*-test (significance at *I^2^*> 50%). Begg’s test, a funnel plot analysis and Egger’s linear regression analysis were conducted to calculate the publication bias. *P*-value <0.10 was considered as statistically significant in both Begg’s or Egger’s test. What is more, sensitivity analyses were performed to inspect whether the summary findings were robust after excluding one or two outlying studies.

## Results

### Characteristics of the eligible studies

**Figure 1:**
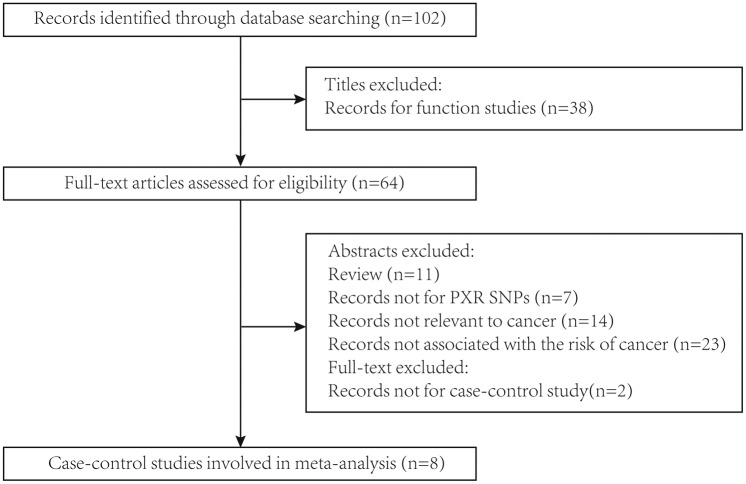
The flow chart of identification for studies included in the meta-analysis

According to the selection process showed in Figure 1, total 102 publications were collected through database searching. Ninety-five records were excluded after reading titles and abstracts (38 were functional studies; 11 were reviews; 2 were not case–control studies; 7 were not related to *PXR* SNPs; 14 were not correlated with cancer; 23 were not associated with cancer risks). Hence, total eight case–control studies (from seven citations) covering 11143 cases and 12170 controls were involved in our meta-analyses, which met the inclusion criteria and the quality assessment. Moreover, the genotype distributions of all records were in agreement with HWE (*P*_HWE_>0.05). The characteristics of these included articles were shown in Table 1 and the distributions of *PXR* SNPs genotype frequency were reported in Table 2.

**Table 1:** The main features of enrolled studies

Ref. No.	Year	Country	Ethnicity	Sample size		Source of controls	Genotyping method	Adjusted factors	Quality score	Citation
				Case	Control					
1	2008	China/Malay/Indian	Asian	62	300	PB	Applied Biosystems 3730 DNA Analyzer	NM	7	[5]
2	2010	Germany	Asian	2984	5318	PB	MALDI-TOF MS	Age, study region, family history of breast cancer, and BMI	10	[21]
3	2011	Germany	Caucasian	1020	1014	PB	MALDI-TOF MS	Age, menopausal status, family history of breast cancer, body mass index, and smoking	8.5	[13]
4	2011	Germany	Caucasian	678	669	PB	KASPar assays	Age, sex, body mass index, and physical activity in METs	6.5	[22]
5	2014	China	Asian	1056	1056	HB	TaqMan	Age and gender	8	[4]
6	2014	China	Asian	503	623	HB	TaqMan	Age and gender	8	[4]
7	2014	Mexican	Mixed	99	144	HB	TaqMan	Age and marital status	6.5	[23]
8	2015	China	Asian	1033	1147	HB	MALDI-TOF MS	Age, sex, BMI, and family history of cancer	8	[24]

Abbreviations: HB, hospital based; KASPar assay, KBioscience’s competitive allele-specific PCR amplification; MALDI-TOF MS, matrix-assisted laser desorption/ionization time-of-flight mass spectrometry using the Sequenom MassARRAY platform and iPLEX GOLD methodology; NM, not mentioned; PB, population based.

**Table 2:** Genotype frequency distributions of PXR SNPs in included studies

Ref. No.	Year	Cancer type	SNPs^1^	Sample size		Case			Control			*P*_HWE_	Included in meta-analysis
				Case	Control	Homozygote wild	Heterozygote	Homozygote variant	Homozygote wild	Heterozygote	Homozygote variant		
1	2008	Breast cancer	rs3814055 (C/T)	62	300	36	23	3	176	106	18	0.702	Yes
		Breast cancer	rs1523127 (A/C)	62	300	36	24	2	170	107	23	0.289	Yes
		Breast cancer	rs2276706 (G/A)	62	300	37	23	2	176	105	19	0.533	Yes
		Breast cancer	rs3732358 (G/A)	62	300	62	0	0	295	5	0	0.884	No^3^
		Breast cancer	rs3732359 (A/G)	62	300	11	28	23	101	125	74	**0.006**	No^2,3^
		Breast cancer	rs3732360 (C/T)	62	300	11	28	23	102	124	74	**0.004**	No^2,3^
		Breast cancer	rs6438550 (A/G)	62	300	50	12	0	216	76	8	0.674	No^3^
		Breast cancer	rs3814057 (A/C)	59	300	18	27	14	125	127	48	0.105	Yes
		Breast cancer	rs3814058 (C/T)	59	300	18	27	14	125	127	48	0.105	Yes
2	2010	Breast cancer	rs6785049 (A/G)	2984	5318	1176	1382	426	2036	2476	806	0.238	Yes
		Breast cancer	rs10504191 (G/A)	2982	5315	2216	713	53	3942	1260	113	0.297	Yes
3	2011	Colorectal cancer	rs1523127 (A/C)	663	669	258	317	88	245	326	98	0.534	Yes
		Colorectal cancer	rs2276706 (G/A)	674	675	267	324	83	251	329	95	0.438	Yes
		Colorectal cancer	rs1464603 (A/G)	676	678	307	291	78	303	310	65	0.263	Yes
		Colorectal cancer	rs6785049 (A/G)	678	677	264	313	101	260	323	94	0.692	Yes
		Colorectal cancer	rs2276707 (C/T)	653	647	439	190	24	446	180	21	0.588	Yes
		Colorectal cancer	rs10504191 (G/A)	673	677	518	143	12	499	161	17	0.356	Yes
		Colorectal cancer	rs3814057 (A/C)	665	657	440	201	24	458	177	22	0.341	Yes
4	2011	Breast cancer	rs3814055 (C/T)	1020	1014	383	487	150	384	497	133	0.159	Yes
		Breast cancer	rs1523127 (A/C)	1020	1013	386	479	155	390	483	140	0.623	Yes
		Breast cancer	rs2276706 (G/A)	1020	1014	388	482	150	400	485	129	0.336	Yes
		Breast cancer	rs1464603 (A/G)	1019	1013	484	446	89	467	451	95	0.352	Yes
		Breast cancer	rs6785049 (A/G)	1020	1012	421	471	128	391	486	135	0.406	Yes
		Breast cancer	rs2276707 (C/T)	1018	1013	682	310	26	690	292	31	0.987	Yes
		Breast cancer	rs10504191 (G/A)	1020	1013	767	235	18	754	239	20	0.835	Yes
		Breast cancer	rs3814057 (A/C)	1020	1009	687	308	25	703	277	29	0.786	Yes
5	2014	Lung cancer	rs3814055 (C/T)	1056	1056	693	328	35	706	316	34	0.851	Yes
		Lung cancer	rs3732360 (C/T)	1056	1056	347	520	189	346	533	177	0.242	Yes
		Lung cancer	rs3814058 (C/T)	1056	1056	315	505	236	365	491	200	0.128	Yes
6	2014	Lung cancer	rs3814058 (C/T)	503	623	122	254	127	185	303	135	0.600	Yes
7	2014	Prostate cancer	rs2472677 (T/C)	99	144	40	43	16	50	72	22	0.637	Noc
		Prostate cancer	rs7643645 (G/A)	99	144	21	45	33	26	75	43	0.499	Noc
8	2015	Colorectal cancer	rs3732360 (C/T)	1033	1147	362	519	152	434	560	153	0.189	Yes
		Colorectal cancer	rs3814058 (C/T)	1033	1147	282	511	240	421	561	165	0.318	Yes

Abbreviation: *P*_HWE_, the *P*-value for HWE in control groups. The results are in bold if *P*<0.05. ^1^, The ancestral alleles were referenced in the NCBI database. ^2^, Excluded due to the SNP not being in accordance with HWE. ^3^, Excluded due to the limited number for this locus.

In general, obtained from eight eligible case–control studies, ten SNPs were involved in our final analysis including: rs10504191 G/A, rs3814058 C/T, rs6785049 A/G, rs1464603 A/G, rs1523127 A/C, rs2276706 G/A, rs2276707 C/T, rs3732360 C/T, rs3814055 C/T, rs3814057 A/C. Of these ten SNPs, the most prevalent one was rs3814058 with four articles encompassing 2651 cases and 3123 controls in Asian population. For rs10504191, rs6785049, rs1523127, rs2276706, rs3814055, and rs3814057 polymorphisms, three case–control studies were enrolled. Other polymorphisms were only investigated in two case–control studies.

### Quantitative data synthesis of ten PXR SNPs

We analyzed the associations between each *PXR* SNP and cancer risk, based on the whole population or two subgroup population stratified by ethnicity or cancer type, respectively. The stratified analyses were performed due to the existence of between-study heterogeneity. In whole population analyses, two (rs3814058 and rs3814057) of the ten SNPs were illustrated to be associated with cancer risk, while others did not show remarkable relations. Moreover, in subgroup analyses, seven SNPs (rs10504191, rs3814058, rs6785049, rs1523127, rs2276706, rs3814055 and rs3814057) were analyzed in ‘cancer type’ subgroup and four SNPs (rs1523127, rs2276706, rs3814055, and rs3814057) were analyzed in ‘ethnicity’ subgroup. However, only rs3814058 showed its association in lung cancer subgroup.

### The PXR rs3814058 C/T polymorphism

For rs3814058 C/T, its heterozygote genotype, homozygote variant genotype, dominant, recessive, and allelic models were all correlated with an elevated risk of cancer in Asian population (CT compared with CC: pooled OR = 1.280, 95%CI = 1.134–1.445, *P*=6.36E-05; TT compared with CC: pooled OR = 1.663, 95%CI = 1.268–2.182, *P*=2.40E-04 dominant model: pooled OR = 1.382, 95%CI = 1.233–1.549, *P*=2.58E-08; recessive model: pooled OR = 1.422, 95%CI = 1.132–1.786, *P*=0.002; T compared with C: pooled OR = 1.292, 95%CI = 1.140–1.465, *P*=6.35E-05). Moreover, the same effect could also be found in lung cancer subgroup analysis (CT compared with CC: OR = 1.271, 95%CI = 1.036–1.429, *P*=0.017; TT compared with CC: OR = 1.387, 95%CI = 1.141–1.687, *P*=0.001; dominant model: OR = 1.267, 95%CI = 1.089–1.473, *P*=0.002; recessive model: OR = 1.228, 95%CI = 1.038–1.452, *P*=0.017; T compared with C: OR = 1.186, 95%CI = 1.075–1.308, *P*=0.001, Table 3).

**Table 3:** Meta-analysis of the association between PXR polymorphisms and cancer risk

SNPs	*n*	Heterozygote compared with homozygote wild			Homozygote variant compared with homozygote wild			Dominant model			Recessive model			Allelic model		
		*P*	OR (95%CI)	*I^2^* (%)	*P*	OR (95%CI)	*I^2^* (%)	*P*	OR (95%CI)	*I^2^* (%)	*P*	OR (95%CI)	*I^2^* (%)	*P*	OR (95%CI)	*I^2^* (%)
**rs10504191 (G/A)**	3	0.656	0.980 (0.897–1.071)	0	0.157	0.820 (0.624–1.079)	0	0.441	0.967 (0.887–1.053)	0	0.166	0.825 (0.628–1.083)	0	0.277	0.958 (0.887–1.035)	0
Cancer type																
Breast cancer	2	0.97	0.998 (0.909–1.097)	0	0.259	0.844 (0.629–1.133)	0	0.757	0.986 (0.900–1.080)	0	0.259	0.845 (0.630–1.132)	0	0.549	0.975 (0.899–1.058)	0
Colorectal cancer	1	0.234	0.856 (0.662–1.106)	NA	0.313	0.680 (0.321–1.438)	NA	0.165	0.839 (0.655–1.075)	NA	0.359	0.705 (0.334–1.487)	NA	0.129	0.842 (0.674–1.051)	NA
**rs3814058 (C/T)**	4	**6.36E-05**	1.280 (1.134–1.445)	0	**2.40E-04^1^**	1.663 (1.268–2.182)	62.5	**2.58E-08**	1.382 (1.233–1.549)	4.1	**0.002^1^**	1.422 (1.132–1.786)	60.3	**6.35E-05^1^**	1.292 (1.140–1.465)	56.9
Cancer type																
Lung cancer	2	**0.017**	1.271 (1.036–1.429)	0	**0.001**	1.387 (1.141–1.687)	0	**0.002**	1.267 (1.089–1.473)	0	**0.017**	1.228 (1.038–1.452)	0	**0.001**	1.186 (1.075–1.308)	0
Breast cancer	1	0.237	1.476 (0.774–2.815)		0.074	2.025 (0.934–4.391)	NA	0.112	1.627 (0.893–2.964)	NA	0.154	1.633 (0.832–3.207)	NA	0.055	1.476 (0.992–2.197)	NA
Colorectal cancer	1	0.002	1.360 (1.122–1.649)	NA	<0.001	2.172 (1.693–2.786)	NA	<0.001	1.544 (1.287–1.853)	NA	<0.001	1.801 (1.447–2.243)	NA	<0.001	1.452 (1.287–1.637)	NA
**rs6785049 (A/G)**	3	0.235	0.952 (0.878–1.032)	0	0.188	0.925 (0.825–1.039)	0	0.152	0.946 (0.876–1.021)	0	0.345	0.950 (0.854–1.057)	0	0.133	0.959 (0.908–1.013)	0
Cancer type																
Breast cancer	2	0.262	0.952 (0.873–1.038)	0	0.126	0.908 (0.803–1.027)	0	0.146	0.941 (0.867–1.021)	0	0.228	0.932 (0.832–1.045)	0	0.098	0.952 (0.898–1.009)	0
Colorectal cancer	1	0.692	0.954 (0.757–1.203)	NA	0.736	1.058 (0.762–1.470)	NA	0.84	0.978 (0.786–1.217)	NA	0.596	1.086 (0.801–1.471)	NA	0.898	1.010 (0.865–1.180)	NA
**rs1464603 (A/G)**	2	0.418	0.943 (0.818–1.087)	0	0.904	1.015 (0.799–1.288)	16.8	0.51	0.956 (0.835–1.094)	0	0.698	1.046 (0.833–1.314)	31.9	0.746	0.983 (0.888–1.089)	0
**rs1523127 (A/C)**	3	0.731	0.975 (0.846–1.125)	0	0.872	0.983 (0.800–1.209)	30.9	0.73	0.976 (0.853–1.118)	0	0.99	1.001 (0.827–1.211)	27.9	0.811	0.988 (0.898–1.088)	0
Cancer type																
Breast cancer	2	0.935	1.008 (0.842–1.206)	0	0.605	1.072 (0.825–1.393)	40.9	0.82	1.020 (0.860–1.209)	0	0.561	1.074 (0.844–1.368)	44.9	0.649	1.029 (0.911–1.162)	0
Colorectal cancer	1	0.503	0.923 (0.731–1.166)	NA	0.354	0.853 (0.609–1.194)	NA	0.388	0.907 (0.727–1.132)	NA	0.469	0.892 (0.654–1.216)	NA	0.33	0.925 (0.791–1.082)	NA
Ethnicity																
Caucasian	2	0.685	0.970 (0.838–1.124)	0	0.951	1.007 (0.816–1.241)	34.5	0.76	0.979 (0.851–1.125)	0	0.812	1.024 (0.844–1.242)	19.5	0.925	0.995 (0.902–1.098)	27.8
Asian	1	0.843	1.059 (0.599–1.874)	0	0.241	0.411 (0.093–1.820)	NA	0.84	0.944 (0.543–1.643)	NA	0.224	0.401 (0.092–1.749)	NA	0.495	0.852 (0.538–1.349)	NA
																
**rs2276706 (G/A)**	3	0.859	0.987 (0.857–1.137)	0	0.888	1.015 (0.823–1.253)	46.7	0.915	0.993 (0.868–1.135)	20.7	0.799	1.026 (0.844–1.246)	41	0.96	1.002 (0.910–1.104)	27.3
Cancer type																
Breast cancer	2	0.777	1.026 (0.858–1.227)	0	0.286	1.157 (0.885–1.511)	20.8	0.565	1.051 (0.887–1.246)	0	0.283	1.146 (0.894–1.468)	23.2	0.344	1.061 (0.939–1.199)	0
Colorectal cancer	1	0.512	0.926 (0.735–1.166)	NA	0.258	0.821 (0.584–1.155)	NA	0.359	0.902 (0.725–1.124)	NA	0.34	0.857 (0.625–1.176)	NA	0.261	0.914 (0.782–1.069)	NA
Ethnicity																
Caucasian	2	0.827	0.984 (0.850–1.138)	0	0.972^1^	1.007 (0.696–1.456)	65.2	0.942	0.995 (0.866–1.143)	0	0.893^1^	1.022 (0.746–1.399)	58.8	0.981^1^	0.998 (0.852–1.169)	59.8
Asian	1	0.888	1.042 (0.587–1.849)	NA	0.366	0.501 (0.112–2.243)	NA	0.883	0.959 (0.549–1.674)	NA	0.35	0.493 (0.112–2.173)	NA	0.623	0.890 (0.558–1.418)	NA
**rs2276707 (C/T)**	2	0.356	1.073 (0.924–1.248)	0	0.896	0.974 (0.655–1.449)	0	0.405	1.064 (0.920–1.230)	0	0.813	0.954 (0.643–1.415)	0	0.518	1.042 (0.919–1.182)	0
**rs3732360 (C/T)**	2	0.537	1.043 (0.913–1.190)	0	0.212	1.123 (0.936–1.349)	0	0.346	1.062 (0.937–1.205)	0	0.257	1.100 (0.933–1.298)	0	0.215	1.056 (0.969–1.151)	0
**rs3814055 (C/T)**	3	0.745	1.022 (0.898–1.163)	0	0.431	1.098 (0.870–1.387)	0	0.593	1.034 (0.914–1.171)	0	0.373	1.105 (0.888–1.375)	0	0.421	1.040 (0.945–1.145)	0
Ethnicity																
Asian	2	0.535	1.058 (0.886–1.263)	0	0.951	1.014 (0.647–1.590)	0	0.551	1.054 (0.887–1.251)	0	0.982	0.995 (0.637–1.554)	0	0.615	1.038 (0.897–1.203)	0
Caucasian	1	0.854	0.982 (0.813–1.187)	NA	0.378	1.131 (0.861–1.486)	NA	0.881	1.014 (0.847–1.213)	NA	0.301	1.142 (0.888–1.469)	NA	0.531	1.041 (0.918–1.182)	NA
Cancer type																
Breast cancer	2	0.91	0.990 (0.827–1.184)	0	0.428	1.114 (0.853–1.453)	0	0.866	1.015 (0.856–1.204)	0	0.348	1.125 (0.880–1.439)	0	0.557	1.037 (0.918–1.172)	0
Lung cancer	1	0.558	1.057 (0.877–1.274)	NA	0.847	1.049 (0.647–1.701)	NA	0.55	1.057 (0.882–1.265)	NA	0.903	1.030 (0.638–1.665)	NA	0.579	1.045 (0.895–1.220)	NA
**rs3814057 (A/C)**	3	**0.036**	1.170 (1.010–1.355)	0	0.457	1.145 (0.802–1.634)	32.6	**0.037**	1.162 (1.009–1.339)	0	0.656	1.082 (0.766–1.527)	9.1	0.053	1.127 (0.999–1.271)	8
Ethnicity																
Caucasian	2	0.061	1.155 (0.993–1.343)	0	0.961	0.990 (0.663–1.478)	0	0.081	1.138 (0.984–1.317)	0	0.795	0.948 (0.637–1.412)	0	0.152	1.097 (0.966–1.245)	0
Asian	1	0.237	1.476 (0.774–2.815)	NA	0.074	2.025 (0.934–4.391)	NA	0.112	1.627 (0.893–2.964)	NA	0.154	1.633 (0.832–3.207)	NA	0.055	1.476 (0.992–2.197)	NA
Cancer type																
Breast cancer	2	0.11	1.163 (0.966–1.399)	0	0.556^1^	1.275 (0.567–2.865)	66.3	0.117	1.154 (0.965–1.379)	28.5	0.687^1^	1.141 (0.602–2.160)	54.5	0.25^1^	1.191 (0.884–1.605)	53.6
Colorectal cancer	1	0.173	1.182 (0.929–1.504)	NA	0.674	1.136 (0.627–2.055)	NA	0.167	1.177 (0.934–1.483)	NA	0.796	1.081 (0.600–1.947)	NA	0.201	1.139 (0.933–1.391)	NA

^1^, *P* was calculated by random model. The results are in bold if *P*<0.05.

### The PXR rs3814057 A/C polymorphism

For rs3814057 A/C, its heterozygote genotype and dominant models were found to be correlated with an increased cancer risk in whole population (AC compared with AA: pooled OR = 1.170, 95%CI = 1.010–1.355, *P*=0.036; dominant model: pooled OR = 1.162, 95%CI = 1.009–1.339, *P*=0.037, Table 3). No association of rs3814057 was found in other genetic models or any subgroups analysis (Table 3).

### Sensitivity analysis

Sensitivity analyses were performed to investigate the influence of individual study on the pooled findings by calculating the sensitivity before and after excluding each study from the meta-analysis (Supplementary Table S1). For rs3814057, it was no longer significant after the removal of each study individually (Supplementary Table S1).

### Publication bias

Begg’s tests and Egger’s tests were used to calculate the potential publication bias. Evaluation of publication bias for all meta-analyses revealed that the publication biases were observed in rs3814055 (the variant genotype and the recessive model) and in rs3814057 (all models), for *P*<0.1 in Egger’s tests (Table 4). This may be caused by language bias, the insufficiency publications with adverse results and/or the elevated estimates due to a deficient methodological design for small studies [25].

**Table 4:** The results of Begg’s and Egger’s tests for the publication bias

Comparison type	Begg’s test		Egger’s test	
	*Z* value	*P*-value	*t* value	*P*-value
**rs10504191 (G/A)**				
Heterozygote compared with homozygote wild	−1.570	0.117	−2.130	0.279
Homozygote variant compared with homozygote wild	−0.520	0.602	−0.550	0.682
Dominant model	−1.570	0.117	−1.800	0.323
Recessive model	−0.520	0.602	−0.420	0.749
Allelic model	−1.570	0.117	−1.530	0.368
**rs3814058 (C/T)**				
Heterozygote compared with homozygote wild	0.000	1.000	0.570	0.629
Homozygote variant compared with homozygote wild	0.680	0.497	0.120	0.912
Dominant model	0.000	1.000	0.300	0.795
Recessive model	0.680	0.497	0.070	0.949
Allelic model	0.000	1.000	0.150	0.893
**rs6785049 (A/G)**				
Heterozygote compared with homozygote wild	−0.520	0.602	−0.860	0.549
Homozygote variant compared with homozygote wild	0.520	0.602	0.580	0.667
Dominant model	−0.520	0.602	−0.270	0.832
Recessive model	1.570	0.117	1.020	0.495
Allelic model	0.520	0.602	0.280	0.829
**rs1464603 (A/G)**				
Heterozygote compared with homozygote wild	−1.000	0.317	NA	NA
Homozygote variant compared with homozygote wild	1.000	0.317	NA	NA
Dominant model	1.000	0.317	NA	NA
Recessive model	1.000	0.317	NA	NA
Allelic model	1.000	0.317	NA	NA
**rs1523127 (A/C)**				
Heterozygote compared with homozygote wild	−0.520	0.602	0.270	0.830
Homozygote variant compared with homozygote wild	−1.570	0.117	−1.410	0.392
Dominant model	−0.520	0.602	−0.390	0.761
Recessive model	−1.570	0.117	−1.670	0.343
Allelic model	−0.520	0.602	−0.870	0.543
**rs2276706 (G/A)**				
Heterozygote compared with homozygote wild	−0.520	0.602	0.050	0.967
Homozygote variant compared with homozygote wild	−0.520	0.602	−0.840	0.556
Dominant model	−0.520	0.602	−0.350	0.785
Recessive model	−0.520	0.602	−0.940	0.521
Allelic model	−0.520	0.602	−0.580	0.668
**rs2276707 (C/T)**				
Heterozygote compared with homozygote wild	−1.000	0.317	NA	NA
Homozygote variant compared with homozygote wild	1.000	0.317	NA	NA
Dominant model	1.000	0.317	NA	NA
Recessive model	1.000	0.317	NA	NA
Allelic model	1.000	0.317	NA	NA
**rs3732360 (C/T)**				
Heterozygote compared with homozygote wild	−1.000	0.317	NA	NA
Homozygote variant compared with homozygote wild	1.000	0.317	NA	NA
Dominant model	−1.000	0.317	NA	NA
Recessive model	1.000	0.317	NA	NA
Allelic model	−1.000	0.317	NA	NA
**rs3814055 (C/T)**				
Heterozygote compared with homozygote wild	−0.520	0.602	0.230	0.857
Homozygote variant compared with homozygote wild	−1.570	0.117	−25.410	**0.025**
Dominant model	0.520	0.602	−0.100	0.939
Recessive model	−1.570	0.117	−9.210	**0.069**
Allelic model	−0.520	0.602	−2.770	0.220
**rs3814057 (A/C)**				
Heterozygote compared with homozygote wild	1.570	0.117	10.860	**0.058**
Homozygote variant compared with homozygote wild	1.570	0.117	8.400	**0.075**
Dominant model	1.570	0.117	11.800	**0.054**
Recessive model	1.570	0.117	52.120	**0.012**
Allelic model	1.570	0.117	13.760	**0.046**

Abbreviation: NA, not available. The results are in bold if *P*<0.1.

### TSA and FPRP analyses

**Figure 2:**
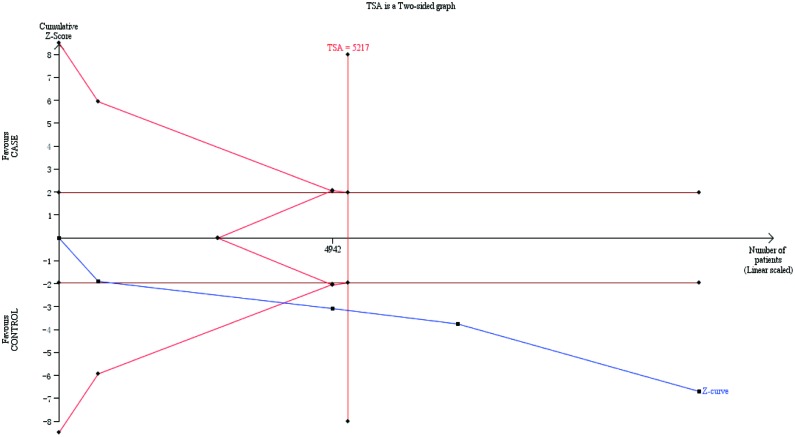
The required information size to demonstrate the relevance of *PXR* rs3814058 SNP with cancer risk The blue line is the cumulative Z-curve. The red inward-sloping line represents the trial sequential monitoring boundaries.

To prevent random errors and intensify the reliability of our conclusions, we conducted TSA. Regarding the rs3814058 SNP, its TSA analysis elucidated that the cumulative evidence for rs3814058 SNP is adequate and no further trials are needed to reinforce our conclusions (Figure 2). For other SNPs, however, TSA analysis showed that there was no sufficient cumulative evidence to strengthen the robustness of our findings (figures were not shown).

Finally, we computed the FPRP values for significant findings. With the assumption of prior probability 0.1, the FPRP values (for all genotype models in overall cancer analysis and the heterozygote genotype, homozygote variant genotype and dominant models in lung cancer subgroup analysis) of rs3814058 SNP were <0.20, implying that these significant correlations were noteworthy (Table 5). On the contrary, none of the FPRP values of rs3814057 SNP were <0.20 (Table 5).

**Table 5:** FPRP values for correlations between genotype frequency of PXR and cancer risk

Genotype	OR (95%CI)	*P*-value	Statistical power^1^	Prior probability^3^				
				0.25	0.1	0.01	0.001	0.0001
**rs3814058 (C/T)**								
CT compared with CC	1.280 (1.134–1.445)	6.36E-05	0.599	**0.000**	**0.001**	**0.010**	0.096	0.515
TT compared with CC	1.674 (1.262–2.219)	3.45E-04	1.000	**0.001**	**0.003**	**0.033**	0.256	0.775
CT + TT compared with CC	1.382 (1.233–1.549)	2.58E-08	0.319	**0.000**	**0.000**	**0.000**	**0.000**	**0.001**
TT compared with CT + CC	1.422 (1.132–1.786)	0.002	0.974	**0.006**	**0.018**	**0.169**	0.672	0.954
T compared with C	1.292 (1.140–1.465)	6.35E-05	0.657	**0.000**	**0.001**	**0.009**	**0.088**	0.491
Subgroup (lung cancer)								
CT compared with CC	1.271 (1.036–1.429)	0.017	0.802	**0.060**	**0.160**	0.677	0.955	0.995
TT compared with CC	1.387 (1.141–1.687)	0.001	0.480	**0.006**	**0.018**	**0.171**	0.676	0.954
CT + TT compared with CC	1.267 (1.089–1.473)	0.002	0.223	**0.026**	**0.075**	0.470	0.900	0.989
TT compared with CT + CC	1.228 (1.038–1.452)	0.017	0.173	0.228	0.470	0.907	0.990	0.999
T compared with C	1.186 (1.075–1.308)	0.001	0.000^2^	0.968	0.989	0.999	1.000	1.000
**rs3814057 (A/C)**								
AC compared with AA	1.170 (1.010–1.355)	0.036	0.297	0.267	0.522	0.923	0.992	0.999
AC + CC compared with AA	1.162 (1.009–1.339)	0.037	0.300	0.270	0.526	0.924	0.992	0.999

^1^, Statistical power was computed using the sample size of case and control, OR and *P-*values. ^2^, When the statistical power<0.0001, we regarded it as 0.0001. ^3^, The FPRP are in bold if the values are <0.2.

## Discussion

Through numerous mechanisms, *PXR* have been revealed to regulate cell proliferation in a plenty of cancers, including colon, liver, breast, prostate, ovarian, and so on [26]. It is widely accepted that the polymorphisms of *PXR* might be correlated to the predisposition to cancer by influencing its expression and/or its function. In the present study, we gathered all related case–control studies and available data, presenting the first systematic review and meta-analysis for the association between ten prevalently studied SNPs in *PXR* and the susceptibility to overall cancer. Of these ten SNPs, two (rs3814058 C/T and rs3814057 A/C) were demonstrated to be associated with an elevated risk of cancer. No correlations were identified amongst other SNPs.

Our study have generalized the current status of the studies on cancer associated SNPs in *PXR*. In order to reinforce our conclusions, we performed the TSA and FPRP analysis, which could minimize the errors and guide future researchers to decide whether to continue focussing on this topic. What is more, we provided clues for researchers to figure out the complicated mechanisms of cancer development and screen novel functional genetic biomarkers for cancer prediction.

For rs3814058 C/T polymorphism, our study elucidated that it was statistically associated with overall cancer risk in every genotype model and it could also reach the significance in lung cancer subgroup and the significant associations were confirmed by TSA and FPRP. The meta-analysis of rs3814058 covered four case–control studies and three of them reported the same findings with us. Edwin Sandanaraj’s research on breast cancer, however, holds a different attitude. To explain the discrepancy, we observed that the expression of *PXR* was depressed or lost in CRC and lung cancer, however elevated in breast cancer. [3,26–28]. Most likely, this tissue specificity can explain the unconformity of the results and more stratification analysis of cancer type ought to be done for rs3814058 polymorphism. Located in the 3′-UTR region of *PXR*, the C to T transition of rs3814058 obtained a novel miRNA (hsa-*miR-129-5p*) binding site which was identified by bioinformatics analysis, leading to a depression of *PXR* expression level in CRC and lung cancer [4,24]. This could reasonably explain the association between the rs3814058 polymorphism and the increment of cancer susceptibility. Therefore, further researchers should pay more attention to the role of rs3814058 on cancerogenesis.

Regarding rs3814057 A/C polymorphism, our results conflicted with other involved studies to some extent. We revealed that the heterozygote genotype and the dominant models of rs3814057 could elevate the risk of overall cancer, which provided a feasible biomarker for cancer prediction. The meta-analysis of rs3814057 involved three case–control studies. None of them were reported to be associated with cancer risk. Based on the TSA, we noticed that the cumulative evidence of rs3814057 was not adequate enough to obtain a reliable conclusion. Likewise, rs3814057 polymorphism was located in 3′-UTR region of *PXR*, putatively binding to several miRNAs, which was speculated by bioinformatics website ‘https://snpinfo.niehs.nih.gov/’. Thus, the rs3814057 polymorphism might influence the expression of *PXR* gene and boost the tumor progression. The unfortunate reality is that no studies have focussed on the mechanisms of rs3814057 polymorphism thus far. As a consequence, association studies and mechanism studies concentrated on rs3814057 are extremely needed to further confirm its role on cancer prediction.

Limitations in our study must be recognized. First, articles in English rather than in other languages were selected, which might result in publication bias. Second, studies of *PXR* polymorphisms on cancer susceptibility field remains emerging, so that the relevant investigations are limited. Last but not least, though *PXR* gene can influence the development of a variety of cancers, its mechanisms in different cancers have been proved to be distinct [26]. Hence, the tissue specificity must be well recognized in the future studies and meta-analyses of *PXR* polymorphisms focussed on only one cancer are in demand.

In conclusion, we systematically reviewed the association between *PXR* polymorphisms and risk of overall cancer. All available data was obtained to conduct a meta-analysis for ten prevalent SNPs. Two of them (rs3814058 C/T and rs3814057 A/C) were elucidated to be correlated with cancer risk in the whole population or some subgroups. Our study generalized the current status of the studies on cancer associated SNPs in *PXR* gene, providing novel clues for further investigators to identify more biomarkers with cancer-forewarning function.

## Supporting information

**Table S1.** ORs (95%CIs) of sensitivity analysis

## Abbreviations

FPRP: false-positive report probability
HWE: Hardy–Weinberg equilibrium
NR1I2: nuclear receptor subfamily 1 group I member 2
OR: odds ratio
PXR: pregnane X receptor
SNP: single nucleotide polymorphism
SXR: steroid and xenobiotic receptor
TSA: trial sequential analysis
95%CI: 95% confidence interval
