# Supplementary material for: Association between PXR polymorphisms and cancer risk: a systematic review and meta-analysis
Source: Biosci Rep. 2018 Jun 12;38(3):BSR20171614. doi: 10.1042/BSR20171614 (PMC5997801; doi:10.1042/BSR20171614)
Supplement: Supplementary file 1 [file bsr20171614_Supp1.pdf]

Table S1. ORs (95%CIs) of sensitivity analysis

| Excluding literature one by one | Heterozygote vs. Homozygote wild | Homozygote variant vs. Homozygote wild | Dominant model | Recessive model | Allelic model |
|---|---|---|---|---|---|
| | OR (95%CI) | OR (95%CI) | OR (95%CI) | OR (95%CI) | OR (95%CI) |
| **rs10504191 (G/A)** | | | | | |
| Overall | 0.980(0.897-1.071) | 0.820(0.624-1.079) | 0.967(0.887-1.053) | 0.825(0.628-1.083) | 0.958(0.887-1.035) |
| Christina Justenhoven | 0.983(0.892-1.084) | 0.807(0.596-1.093) | 0.968(0.881-1.065) | 0.811(0.600-1.096) | 0.958(0.880-1.043) |
| Sascha Abbas | 0.921(0.785-1.082) | 0.790(0.458-1.287) | 0.910(0.779-1.064) | 0.806(0.496-1.311) | 0.911(0.792-1.047) |
| Anja Rudolph | 0.998(0.909-1.097) | 0.844(0.629-1.133) | 0.986(0.900-1.080) | 0.845(0.630-1.132) | 0.975(0.899-1.058) |
| **rs3814058 (C/T)** | | | | | |
| Overall | 1.280(1.134-1.445) | 1.663(1.268-2.182) | 1.382(1.233-1.549) | 1.422(1.132-1.786) | 1.292(1.140-1.465) |
| Lisha Zhang | 1.339(1.147-1.563) | 1.829(1.339-2.498) | 1.478(1.277-1.710) | 1.522(1.135-2.040) | 1.351(1.170-1.561) |
| Lisha Zhang | 1.283(1.122-1.467) | 1.766(1.221-2.554) | 1.397(1.231-1.584) | 1.508(1.117-2.035) | 1.330(1.124-1.575) |
| Edwin Sandanaraj | 1.274(1.126-1.441) | 1.627(1.196-2.215) | 1.373(1.223-1.542) | 1.401(1.080-1.818) | 1.276(1.109-1.468) |
| Haizhen Ni | 1.231(1.053-1.439) | 1.419(1.174-1.715) | 1.287(1.112-1.489) | 1.248(1.061-1.470) | 1.201(1.092-1.321) |
| **rs6785049 (A/G)** | | | | | |
| Overall | 0.952(0.878-1.032) | 0.925(0.825-1.039) | 0.946(0.876-1.021) | 0.950(0.854-1.057) | 0.959(0.908-1.013) |
| Christina Justenhoven | 0.965(0.882-1.055) | 0.935(0.824-1.061) | 0.957(0.880-1.042) | 0.954(0.848-1.072) | 0.966(0.909-1.026) |
| Sascha Abbas | 0.921(0.796-1.065) | 0.951(0.769-1.176) | 0.928(0.808-1.065) | 0.994(0.816-1.210) | 0.961(0.871-1.060) |
| Anja Rudolph | 0.952(0.873-1.038) | 0.908(0.803-1.027) | 0.941(0.867-1.021) | 0.932(0.832-1.045) | 0.952(0.898-1.009) |
| **rs1464603 (A/G)** | | | | | |
| Overall | 0.943(0.818-1.087) | 1.015(0.799-1.288) | 0.956(0.835-1.094) | 1.046(0.833-1.314) | 0.983(0.888-1.089) |

| | | | | | |
|---|---|---|---|---|---|
| Christina Justenhoven | 0.926(0.740-1.161) | 1.184(0.822-1.707) | 0.971(0.784-1.203) | 1.230(0.869-1.742) | 1.028(0.876-1.207) |
| Anja Rudolph | 0.954(0.795-1.145) | 0.904(0.659-1.240) | 0.945(0.794-1.125) | 0.925(0.683-1.252) | 0.954(0.835-1.089) |
| **rs1523127 (A/C)** | | | | | |
| Overall | 0.975(0.846-1.125) | 0.983(0.800-1.209) | 0.976(0.853-1.118) | 1.001(0.827-1.211) | 0.988(0.898-1.088) |
| Christina Justenhoven | 0.942(0.759-1.169) | 0.813(0.587-1.127) | 0.912(0.743-1.121) | 0.851(0.630-1.150) | 0.917(0.791-1.063) |
| Edwin Sandanaraj | 0.970(0.838-1.124) | 1.007(0.816-1.241) | 0.979(0.851-1.125) | 1.024(0.844-1.242) | 0.995(0.902-1.098) |
| Anja Rudolph | 1.008(0.842-1.206) | 1.072(0.825-1.393) | 1.020(0.860-1.209) | 1.074(0.844-1.368) | 1.029(0.911-1.162) |
| **rs2276706 (G/A)** | | | | | |
| Overall | 0.987(0.857-1.137) | 1.015(0.823-1.253) | 0.993(0.868-1.135) | 1.026(0.844-1.246) | 1.002(0.910-1.104) |
| Christina Justenhoven | 0.941(0.760-1.166) | 0.797(0.572-1.110) | 0.910(0.742-1.116) | 0.832(0.612-1.131) | 0.912(0.786-1.057) |
| Edwin Sandanaraj | 0.984(0.850-1.138) | 1.034(0.835-1.279) | 0.995(0.866-1.143) | 1.043(0.856-1.270) | 1.008(0.914-1.112) |
| Anja Rudolph | 1.026(0.858-1.227) | 1.157(0.885-1.511) | 1.051(0.887-1.246) | 1.146(0.894-1.468) | 1.061(0.939-1.199) |
| **rs2276707 (C/T)** | | | | | |
| Overall | 1.073(0.924-1.248) | 0.974(0.655-1.449) | 1.064(0.920-1.230) | 0.954(0.643-1.415) | 1.042(0.919-1.182) |
| Christina Justenhoven | 1.072(0.841-1.367) | 1.161(0.637-2.116) | 1.082(0.857-1.366) | 1.137(0.627-2.064) | 1.076(0.880-1.316) |
| Anja Rudolph | 1.074(0.887-1.301) | 0.849(0.499-1.444) | 1.052(0.874-1.267) | 0.830(0.489-1.409) | 1.021(0.869-1.200) |
| **rs3732360 (C/T)** | | | | | |
| Overall | 1.043(0.913-1.190) | 1.123(0.936-1.349) | 1.062(0.937-1.205) | 1.100(0.933-1.298) | 1.056(0.969-1.151) |
| Lisha Zhang | 1.111(0.925-1.335) | 1.191(0.914-1.551) | 1.128(0.947-1.344) | 1.121(0.880-1.428) | 1.092(0.966-1.233) |
| Haizhen Ni | 0.973(0.803-1.178) | 1.065(0.826-1.372) | 0.996(0.830-1.194) | 1.083(0.864-1.356) | 1.022(0.904-1.154) |

| | | | | | |
|---|---|---|---|---|---|
| **rs3814055 (C/T)** | | | | | |
| Overall | 1.022(0.898-1.163) | 1.098(0.870-1.387) | 1.034(0.914-1.171) | 1.105(0.888-1.375) | 1.040(0.945-1.145) |
| Lisha Zhang | 0.990(0.827-1.184) | 1.114(0.853-1.453) | 1.015(0.856-1.204) | 1.125(0.880-1.439) | 1.037(0.918-1.172) |
| Edwin Sandanaraj | 1.020(0.893-1.164) | 1.110(0.875-1.408) | 1.035(0.911-1.175) | 1.117(0.894-1.396) | 1.043(0.945-1.150) |
| Christina Justenhoven | 1.058(0.886-1.263) | 1.014(0.647-1.590) | 1.054(0.887-1.251) | 0.995(0.637-1.554) | 1.038(0.897-1.203) |
| **rs3814057 (A/C)** | | | | | |
| Overall | 1.170(1.010-1.355) | 1.145(0.802-1.634) | 1.162(1.009-1.339) | 1.082(0.766-1.527) | 1.127(0.999-1.271) |
| Christina Justenhoven | 1.215(0.970-1.522) | 1.395(0.870-2.236) | 1.230(0.991-1.525) | 1.280(0.818-2.002) | 1.199(1.003-1.434) |
| Edwin Sandanaraj | 1.155(0.993-1.343) | 0.990(0.663-1.478) | 1.138(0.984-1.317) | 0.948(0.637-1.412) | 1.097(0.966-1.245) |
| Anja Rudolph | 1.163(0.966-1.399) | 1.150(0.737-1.793) | 1.154(0.965-1.379) | 1.082(0.707-1.657) | 1.120(0.962-1.302) |

Note: OR, odds ratio; CI, confidence interval.
